# Astaxanthin attenuates glucose-induced liver injury in largemouth bass: role of p38MAPK and PI3K/Akt signaling pathways

**DOI:** 10.1186/s13578-024-01304-7

**Published:** 2024-09-19

**Authors:** Zhihong Liao, Xuanshu He, Anqi Chen, Jian Zhong, Sihan Lin, Yucai Guo, Xin Cui, Baoyang Chen, Wei Zhao, Jin Niu

**Affiliations:** 1 State key Laboratory of Biocontrol, Guangdong Provincial Key Laboratory for Aquatic Economic Animals and Southern Marine Science and Engineering Guangdong Laboratory (Zhuhai), School of Life Sciences, Sun Yat-Sen University, Guangzhou, China; 2 Zhanjiang Customs, Zhanjiang, China

**Keywords:** Astaxanthin, Largemouth bass, Liver injury, Apoptosis, Insulin resistance

## Abstract

**Background:**

Astaxanthin (ASX) has been documented to exert beneficial influence on various processes in fish. Largemouth bass (*Micropterus salmoides*) serves as a common model for studying glucose-induced liver disease, making it imperative to investigate the regulatory mechanisms underlying its liver health.

**Methods:**

Largemouth bass were fed with a control diet (CON), a high carbohydrate diet (HC), or a HC diet supplemented astaxanthin (HCA) for 8-weeks, followed by the glucose tolerance test (GTT). Primary hepatocytes were treated with low glucose and high glucose combined with different concentrations of astaxanthin for 48 h. The histopathology, enzymology, transcriptomics, molecular biology and cell biology were combined to investigate the mechanism of liver injury.

**Results:**

This study provides evidence for the protective effects of ASX against growth performance reduction and hepatic liver injure in largemouth bass fed HC diet. In GTT, HCA diet exhibited an improvement in glucose tolerance following glucose loading. Although HCA diet did not restore the expression of insulin resistance-related genes in livers at different time during the GTT, the addition of ASX in the long-term HC diet did improve the insulin resistance pathway by regulating the PTP1B/PI3K/Akt signaling pathway. Hepatic transcriptome analyses showed that ASX plays an essential role in the modulation of glucose homeostasis in response to treated with HC diet. In in vitro study, ASX treatment resulted in an exaltation in cell viability and a reduction in the rate of cell apoptosis and reactive oxygen species (ROS). Additionally, astaxanthin was observed to improve apoptosis induced by high-glucose via p38MAPK/bcl-2/caspase-3 signaling pathway.

**Conclusions:**

Astaxanthin exhibited a protective effect against apoptosis by regulating p38MAPK/bcl-2/caspase-3 pathway, and ameliorated insulin resistance by activating the PTP1B/PI3K/Akt pathway. This study elucidated the mechanism of astaxanthin in the liver injury of largemouth bass from a new perspective and provided a new target for the treatment of insulin resistance.

**Graphical Abstract:**

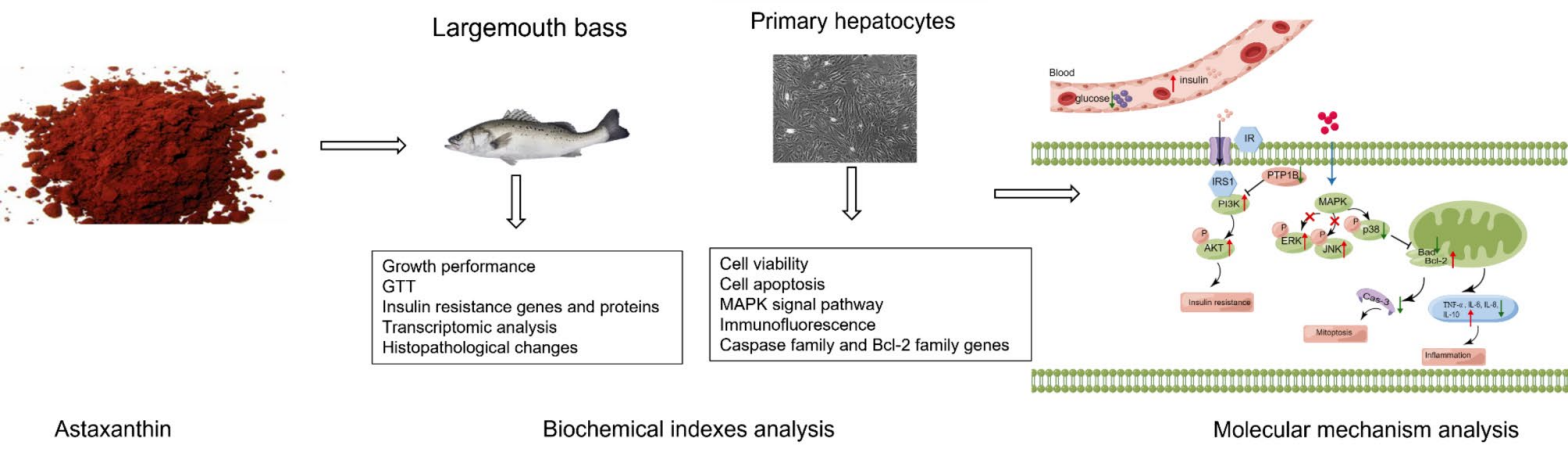

**Supplementary Information:**

The online version contains supplementary material available at 10.1186/s13578-024-01304-7.

## Highlights

ASX improved liver damage in diabetic largemouth bass.

ASX improved apoptosis via p38MAPK/bcl-2/caspase-3 signaling pathway.

ASX alleviated insulin resistance through the PTP1B/PI3K/Akt signaling pathway.

**Supplementary Information:**

The online version contains supplementary material available at 10.1186/s13578-024-01304-7.

## Introduction

Given the increasing scarcity of fish meal protein feed, carbohydrates are now being evaluating as an alternative protein source to fish meal in aquafeed [1, 2]. Unlike mammals utilize carbohydrates preferentially, fish predominantly utilize protein and fat as sources of energy, with a limited ability to metabolize carbohydrates, particularly in carnivorous species [3]. As a typical carnivorous fish model of glucose intolerance, largemouth bass (*Micropterus salmoides*) is widely farmed around the world. There have been extensive studies shown that high dietary carbohydrates increase plasma glucose levels and induces liver injure in largemouth bass, including disorders of metabolism, vacuolation of hepatocytes and fibrosis and accumulation of glycogen [4–7]. Commercial feed formulations for largemouth bass typically contain a maximum of 10% starch to maintain liver health [8]. Although glycogenic hepatopathy appears to be a common disease in carnivorous fish, it has been under-recognized in many studies.

Astaxanthin (ASX) is a potent antioxidant with demonstrated efficacy in ameliorating liver disease [9]. In mammal, astaxanthin has been found to mitigate liver inflammation and fibrosis caused by nonalcoholic steatohepatitis in mice [10]. Studies have also demonstrated the ability of astaxanthin to relieve liver endoplasmic reticulum stress and inflammation in mice fed a diet containing high fructose and high fat [11]. Besides, astaxanthin may be useful in preventing diabetic complications and reversing hepatotoxicity in adult rats [12]. In aquaculture, astaxanthin has been widely adopted for use for enhancing pigmentation and stress resilience. In a prior investigation, we exhibited that astaxanthin enhanced the ability to counteract oxidation, performance in growth, and immune reaction in largemouth bass that were fed a high-fat diet [13]. Collectively, these studies suggested that astaxanthin may represent a novel treatment of dietary-related metabolic disorders not only in mammal but also in fish. Hence, this study is implemented to investigate the potential effect of astaxanthin on the mitigation of high-carbohydrate-induced liver injury in largemouth bass and its possible mechanisms.

The glucose tolerance test (GTT) is widely used to assess the ability of fish to utilize glucose [14, 15]. Carnivorous fish exhibit a notable resistance to insulin and glucose in relation to carbohydrate metabolism, resulting in an elevation of blood glucose concentration with higher starch consumption [16]. Numerous studies have indicated that inadequate insulin secretion is the major cause of such glucose intolerance in fish [17]. In glucose metabolism, insulin first activates the insulin receptor, followed by the PI3K/AKT signaling pathway [18, 19]. The PI3K/AKT pathway is a critical node of insulin signaling [20]. Zhong et al. [5] employed transcriptomic analysis to found that high-carbohydrate diet causes disruption of hepatic glycogen metabolism and liver fibrosis in largemouth bass, which may be mediated by the PI3K/Akt signaling pathway. In mammal, astaxanthin has the ability to ameliorate liver insulin resistance by modulating AMPK and MAPK signaling pathways and enhance post-receptor insulin signaling events by promoting IR-β/PI3K/Akt signal pathway [21, 22]. Nevertheless, the available data on astaxanthin participates in glucose tolerance, insulin sensitivity, and PI3K/Akt signaling pathway to maintain glucose homeostasis in carnivorous fish is scarce.

The objective of this research is to investigate the effects of astaxanthin on high carbohydrate induced insulin resistant and liver damage in largemouth bass. Furthermore, we will delve deeper into the anti-apoptotic properties of astaxanthin on the protein level, which may contribute to develop the nutritional strategies for improving high carbohydrate-induced liver injury.

## Materials and methods

### Experimental diets

Astaxanthin abundance in high-carbohydrate diets was determined from previous studies [23]. As shown in Table 1, three purified isonitrogenous and isolipidic diets were designed and formulated: including a control (CON) diet, a high-carbohydrate (HC) diet and a high-carbohydrate supplemented with 0.1% Lucantin Pink CWD (BASF, Shanghai, China) containing 10% (w/w) astaxanthin (HCA) diet. In all diets, the main source of carbohydrate was corn starch. Bone meal was used for eliminating the difference of quantity caused by corn starch. The experimental diets were conducted using the previously reported method [24]. Further details can be found in Supplemental Methods.

**Table 1:** Ingredients and nutrient composition of the experimental diets (% dry matter)

Ingredients	CON	HC	HCA
Corn starch	0	20	20
Fish meal	45	45	45
Krill meal	3	3	3
Beer yeast	5	5	5
Soybean meal	10.3	10.3	10.2
Wheat gluten	10	10	10
Fish oil	1	1	1
Soy oil	1	1	1
Soya lecithin	1	1	1
Mineral premix^1^	1	1	1
Vitamin premix^2^	1	1	1
Choline chloride (50%)	0.5	0.5	0.5
Monocalcium phosphate	1	1	1
Vitamin C	0.2	0.2	0.2
Bone meal	20	0	0
Lucantin Pink CWD^3^	0	0	0.1
Sum	100	100	100
Nutrient composition			
Crude protein	47.92	48.66	47.90
Crude lipid	6.66	6.68	6.71

^1^Mineral premix provides the following per kg of diet: MgSO_4_∙7H_2_O, 1090 mg; KH_2_PO_4_, 932 mg; NaH_2_PO_4_∙2H_2_O, 432 mg; FeC_6_H_5_O_7_∙5H_2_O, 181 mg; ZnCl_2_, 80 mg; CuSO_4_∙5H_2_O, 63 mg; AlCl_3_∙6H_2_O, 51 mg; MnSO_4_∙H_2_O, 31 mg; KI, 28 mg; CoCl_2_∙6H_2_O, 6 mg; Na_2_SeO_3_∙H_2_O, 0.8 mg

^2^Vitamin premix provides the following per kg of diet: Vitamin B1, 30 mg; Vitamin B2, 60 mg; Vitamin B6, 60 mg; Nicotinic acid, 200 mg; Calcium pantothenate, 100 mg; Inositol, 100 mg; Biotin, 2.5 mg; Folic acid, 10 mg; Vitamin B12, 0.1 mg; Vitamin K3, 40 mg; Vitamin A, 10000IU, Vitamin, 160 IU

^3^Lucantin Pink CWD of 10% (w/w) astaxanthin content provided from BASF, Shanghai, China

### Sample collection

Juvenile largemouth bass were obtained from Shunye Fishery Company (Foshan, China). More details of experiment design and feeding management can be found in Supplemental Methods. Sampling was performed after 8-week feeding trial, all fish were fasted for 24 h prior to sampling. 12 fish from each diet (4 fish per tank) were randomly chosen and measured the body length and weight. In order to prepare serum, caudal vertebral vein blood was sampled using a sterile syringe, then centrifuged at 4000 g for 10 min at 4 °C. The serum was immediately stored at -80 °C to preserve it for future use. For future analyses, the dissected livers were also immediately frozen in liquid nitrogen and then kept at -80 °C. For more details on growth performance and morphology parameters, see Supplemental Methods.

### Glucose tolerance test (GTT)

The GTT method described by Chen et al. [25], blood of largemouth bass from three diet treatment was separately collected from the caudal vein. More details of glucose tolerance test can be found in Supplemental Methods.

### Transcriptomic analysis

Nine liver samples of largemouth bass fed with CON, HC and HCA diets were prepared for transcriptomic analysis. RNA integrity was measured by using the RNA Nano 6000 Assay Kit on the Bioanalyzer 2100 system (Agilent Technologies, CA, USA). More details of transcriptomic analysis can be found in Supplemental Methods.

### Histopathological studies

Liver tissues were fixed in neutral 4% formalin (Servicebio, China) and embedded in paraffin wax. Hematoxylin and eosin (H&E) staining and periodic acid-schiff (PAS) staining were conducted according to the standard protocol. Light microscopy was used to observe and photograph histopathological lesions (NikonNi–U, Nikon Corporation, Tokyo, Japan). For the transmission electron microscopy observations, livers were fixed in 2.5% glutaraldehyde (AAPR46) and rinsed with buffer. To observe the various structures within stained cells, a transmission electron microscope (JEM-1400 Flash, Japan) was used.

### Biochemical analysis

Measurements of serum glucose were performed using glucose oxidase kit (A154-1-1; Nanjing Jiancheng Bioengineering Institute, Nanjing, China). The corresponding reagent kits (C009-2-1 and C010-2-1, respectively; Nanjing Jiancheng Bioengineering Institute, Nanjing, China) were utilized for measuring serum aspartate aminotransferase (AST) and alanine aminotransferase (ALT). The measurement of serum insulin level was conducted with a commercially available Elisa kit (ml0258550; Shanghai Enzyme-linked Biotechnology Co., Ltd., China).

### Western blot and quantitative real-time PCR (RT-PCR)

The livers and cells were used to harvest and extract total protein by utilizing RIPA lysis buffer (FD009; Fdbio science, Hangzhou, China) along with a mixture of protease inhibitor and phosphatase inhibitor cocktail (FD1002; Fdbio science, Hangzhou, China). To measure the amount of total protein, a BCA assay (KS134848; Thermo, Scientific, Waltham, MA, USA) was employed. All details of primary antibodies can be found in Supplemental Methods. The PVDF filters were rinsed and treated with anti-rabbit (SA00001-2; Proteintech, United States, diluted 1:10000) secondary antibody for 1 h at ambient temperature. The Azure 300 ultra-sensitive chemiluminescence imager was utilized to visualize the protein bands. The levels of protein were standardized by β-actin and measured using the Image-Pro Plus software.

The extraction of total RNA and the synthesis of cDNA were performed following the previously described protocol [26]. A gene responsible for maintaining the cleanliness of a house, known as elongation factor 1a (*ef-1α*; GenBank accession no. KT827794), was normalized as an internal reference. Table 2 displays the gene-specific primers utilized for largemouth bass mRNA. The qPCR examination was conducted in a 10 µL reaction volume using a Light Cycler 480II Real-Time System from Roche, located in IN, USA. The qPCR protocol started with a 10 min incubation at 95 °C, followed by 40 cycles consisting of 5 s at 95 °C, 30 s at 60 °C, and 30 s at 72 °C. Additionally, the reaction quality was assessed by analyzing standard melting curves. The 2^−ΔΔCt^ method was used to calculate qPCR data for each sample.

**Table 2:** Sequences of primers used in this study

Genes	Forward primers (5’ to 3’)	Reverse primers (5’ to 3’)	Sources/GenBank No.
*tnf-α*	CTTCGTCTACAGCCAGGCATCG	TTTGGCACACCGACCTCACC	[49]
*il-6*	GACCAGCAGCCAGGAGGA	GGAGGTTGTACACGATGCTG	[49]
*il-8*	CGTTGAACAGACTGGGAGAGATG	AGTGGGATGGCTTCATTATCTTGT	[49]
*il-10*	CGGCACAGAAATCCCAGAGC	CAGCAGGCTCACAAAATAAACATCT	[49]
*cat*	ATCCCTGTGGGCAAAATGGT	CGGTGACGATGTGTGTCTGG	XM_038704976.1
*gsh-px*	GGGGCTCCACCTGCTTCTTG	ACCCCTCTGCTCAGGCATTT	MK614713.1
*sod1*	TGGCAAGAACAAGAACCACA	CCTCTGATTTCTCCTGTCACC	XM_038708943.1
*caspase-3*	GCTTCATTCGTCTGTGTTC	CGAAAAAGTGATGTGAGGTA	[49]
*caspase-8*	GAGACAGACAGCAGACAACCA	TTCCATTTCAGCAAACACATC	[49]
*caspase-9*	CTGGAATGCCTTCAGGAGACGGG	GGGAGGGGCAAGACAACAGGGTG	[49]
*bcl-2*	TGCCTTTGTGGAGCTGTATG	GGAAGAGGAGGAGGAGGATG	[49]
*bax*	TCTTCACTCAGTCCCACAAA	ATACCCTCCCAGCCACC	XM_038704178.1
*bad*	CACATTTCGGATGCCACTAT	TTCTGCTCTTCTGCGATTGA	XM_038730645.1
*ir*	CATTTTGAGGGAACTGGGTC	CTTGATGATGTCTTTAGCGA	[49]
*irs1*	TAGTGGTGGTGTCAGCGGT	GGAGGTGGAAGTAAAGGAT	MT431531
*pi3kr1*	AAGACCTTCCTCATCACGAC	CCTTCCACTACAACACTGCA	Cluster-21914.23096
*ef-1α*	TGCTGCTGGTGTTGGTGAGTT	TTCTGGCTGTAAGGGGGCTC	KT827794.1

### Culture of largemouth bass primary hepatocytes

Largemouth bass primary hepatocytes were isolated and cultured as follows: briefly, the liver was minced as small as possible with surgical scissors under sterile conditions, and washed thoroughly with pre-warmed phosphate-buffered saline (PBS) to remove the blood and other components. The rinsed livers were enzymatically digested using trypsin (25200072; Thermo Fisher Scientific, Waltham, MA, USA) at 28 °C for 40 min. Centrifuge the cells after 6 min at 1000 rpm, discard supernatant, and resuspend harvested cell pellet in low-glucose medium containing 20% FBS and 1% penicillin-streptomycin. The isolated hepatocytes were seeded at a density of 1 × 10^6^ cells/mL and cultured in a humidified 28 °C incubator with 5% CO_2_. When the confluence reached 70–80%, cells were divide into six groups: (1) LG, treated with low-glucose for 48 h; (2) HG, treated with high-glucose for 48 h; (3) HG + 10 µM ASX, treated with 10 µM astaxanthin and high-glucose for 48 h; (4) HG + 20 µM ASX, treated with 20 µM astaxanthin and high-glucose for 48 h; (5) HG + 30 µM ASX, treated with 30 µM astaxanthin and high-glucose for 48 h; (6) HGA, HG + 50 µM ASX, treated with 50 µM astaxanthin and high-glucose for 48 h. Astaxanthin (S3834; Selleck Chemicals, Houston, Texas, USA) was added at the start of low or high glucose culture and remained present throughout the experiment. The cells were pretreated with various concentrations of SB203580 (p38 MAPK pathway inhibitor; S1076, Selleck Chemicals, Houston, Texas, USA) for 2 h, then treated with HG or HGA for 48 h.

### CCK8 assay

Six replicates of primary hepatocytes were seeded in a 96-well culture plate at a density of 1 × 10^4^ cells/mL. Subsequently, the cells were exposed to different concentrations of astaxanthin in combination with a glucose solution. Cell viability was assessed after incubating for either 24–48 h using a CCK8 assay (FD3788; Fdbio science, Hangzhou, China), following the guidelines provided by the manufacturer.

### Annexin V-FITC/PI staining

Flow cytometry was used to examine apoptosis by employing annexin V-FITC/PI staining (BL110A; Biosharp life science, Beijing, China). Cells (1 × 10^6^) were seeded in 6-well plates and exposed to glucose and astaxanthin for 48 h. Afterward, the cells were digested by trypsin without EDTA and washed twice with chilled PBS. Finally, they were suspended in 100 µL of binding buffer. The cells were stained with Annexin V-FITC (5 µL) for 10 min at room temperature, followed by 10 µL PI for 5 min in the dark. Flow cytometry (Backman cytoflex) was used to analyze the cells.

### ROS detection

ROS formation within the cell was identified by utilizing the H2DCF-DA probe (C-2938; Invitrogen™, Waltham, MA, USA), which is a 6-carboxy-2’, 7’-dichlorodihydrofluorescein diacetate, di (acetoxymethyl ester). After being pretreated with LG, HG, and HGA for 48 h, the primary hepatocytes (1 × 10^6^) were collected and resuspended in serum-free DMEM with 15 µM H2DCF-DA. The harvested primary liver cells were incubated at a temperature of 28 °C for a duration of 30 min and subsequently analyzed using flow cytometry (Backman cytoflex).

### Immunofluorescence analysis

Cells were seeded in a 20-mm laser confocal culture dish (cat. no. BDD012035) and treated with LG, HG and HGA for 48 h. More details of immunofluorescence analysis can be found in Supplemental Methods.

### Statistical analysis

To analyze data on serum parameters in the GTT, a two-way ANOVA was employed to examine variations in treatment means considering sampling time, dietary treatments, and their interaction. If there were significant differences (*P* < 0.05) observed in the interaction, each factor was subsequently analyzed individually using one-way analysis of variance (ANOVA). Means ± SEM, calculated from 3 to 6 biological replication, were used to present additional data. The comparison of variables between the two treatments was done by the student’s t-test. *P < 0.05, **P < 0.01 and ***P < 0.001 were established to indicate statistical difference. GraphPad Prism 8.0 (GraphPad, USA) was responsible for creating all visual elements.

## Results

### Astaxanthin improved growth performance in high carbohydrate-fed largemouth bass

**Fig. 1:**
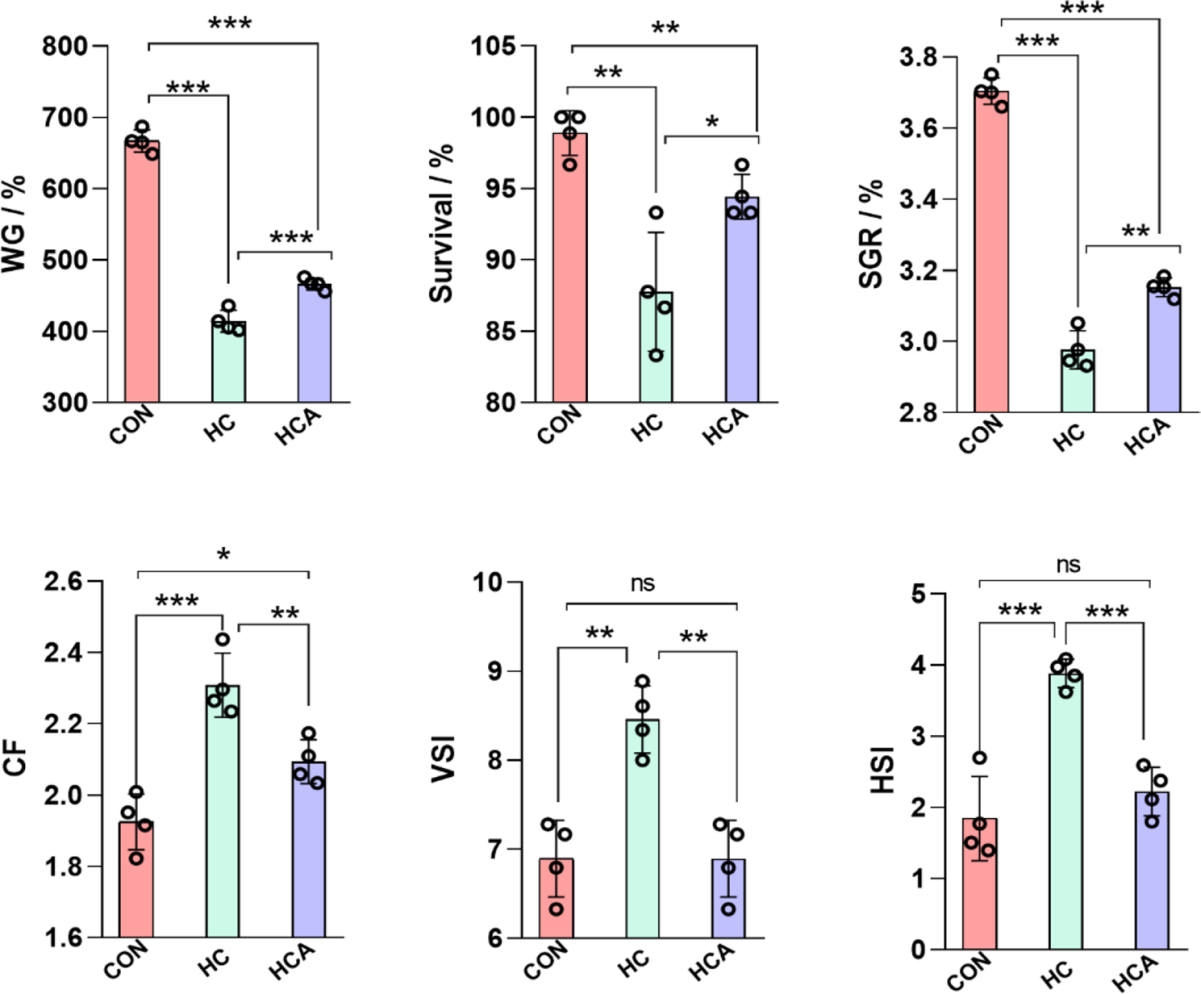
Astaxanthin improved growth performance in high carbohydrate-fed largemouth bass. Values were mean ± SEM of four biological replicates. WG, weigh gain; SGR, specific growth rate; CF, condition factor; VSI, Viscerosomatic index; HSI, hepatosomatic index. *P < 0.05, **P < 0.01 and ***P < 0.001; *ns*, no significant difference

Following an 8-week feeding trial, the impact of astaxanthin on the growth performance of largemouth bass was depicted in Fig. 1. The HC diet exhibited significantly lower WG, survival, and SGR compared to the CON diet, but the inclusion of astaxanthin considerably increased these 3 parameters (*P* < 0.01). CF, VSI and HSI were significantly higher in HC diet than in CON diet (*P* < 0.01). While, the VSI and HSI of largemouth bass fed HCA diet were considerably lower in comparison with those fed the HC diet (*P* < 0.01). These findings collectively demonstrated that astaxanthin supplementation improved the growth performance of largemouth bass that were fed a high-carbohydrate diet.

### Astaxanthin reduced the elevated glucose tolerance and alleviated insulin resistance through the PTP1B/PI3K/Akt signaling pathway

**Fig. 2:**
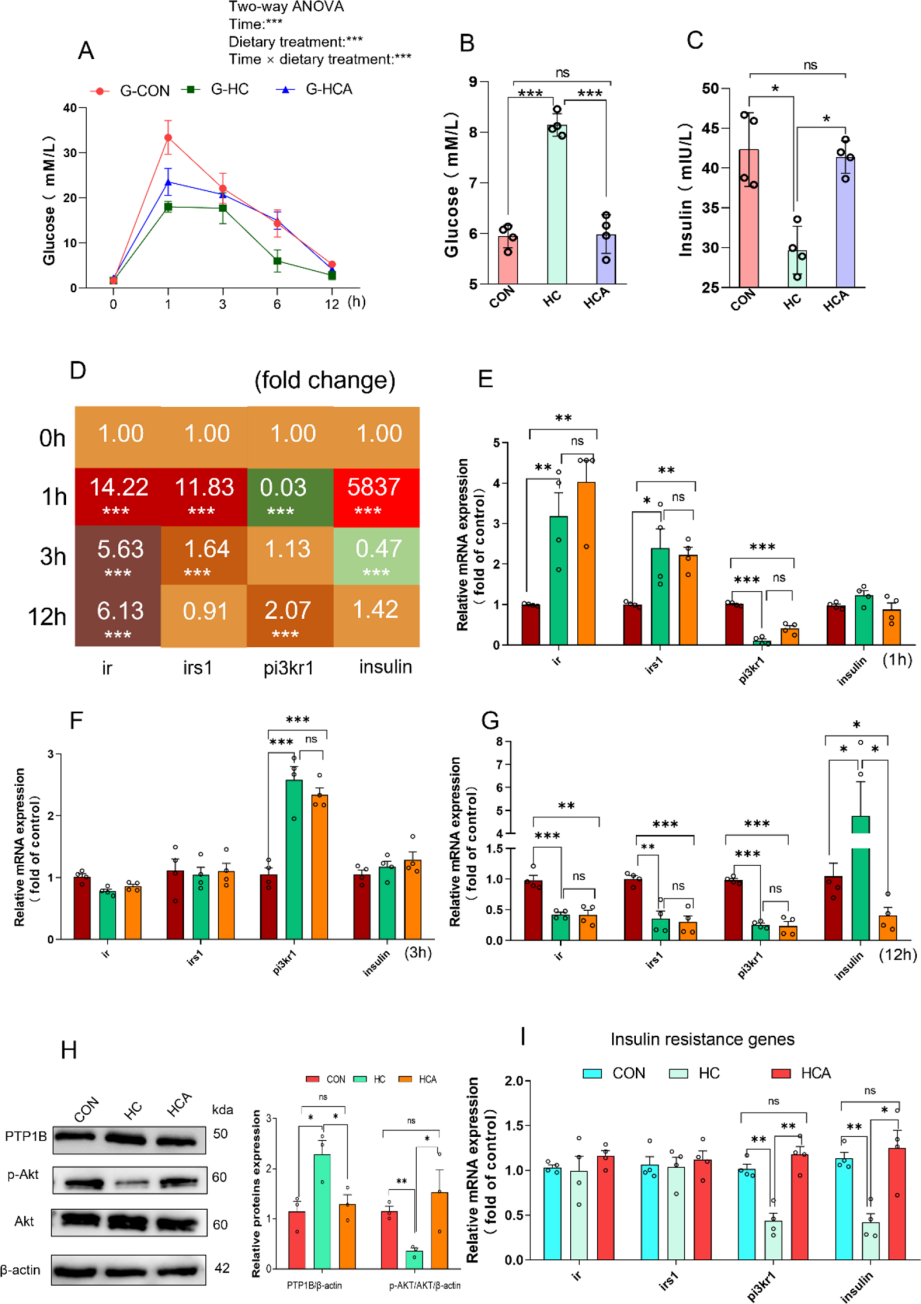
Astaxanthin reduced the elevated glucose tolerance and alleviated insulin resistance through the PTP1B/PI3K/Akt signaling pathway. (**A**) Serum glucose concentration in GTT (*n* = 6). (**B**) The serum glucose level in different diets (*n* = 4). (**C**) The serum insulin level in different diets (*n* = 4). (**D**) Hepatic mRNA fold change of insulin resistance related genes at 0 h, 1 h, 3 h and 12 h after glucose injection (*n* = 6). (**E**) The expression of liver insulin resistance genes (*ir*, *irs1*, *pi3kr1* and *insulin*) of largemouth bass after 1 h of glucose injection (*n* = 4). (**F**) The expression of liver insulin resistance genes (*ir*, *irs1*, *pi3kr1* and *insulin*) of largemouth bass after 3 h of glucose injection (*n* = 4). (**G**) The expression of liver insulin resistance gene (*ir*, *irs1*, *pi3kr1* and *insulin*) of largemouth bass after 12 h of glucose injection (*n* = 4). G-CON: control diet during the GTT; G-HC: high-carbohydrate diet during the GTT; G-HCA: high-carbohydrate diet supplemented with astaxanthin during the GTT. (**H**) The expression of insulin resistance proteins (PTP1B, p-Akt, and Akt) in different diets (*n* = 3). **I** The expression of liver insulin resistance genes (*ir*, *irs1*,* pi3kr1* and *insulin*) in different diets (*n* = 4). Values were mean ± SEM of three-six biological replicates. *P < 0.05, **P < 0.01 and ***P < 0.001; *ns*, no significant difference

To further characterize the glucose homeostasis phenotype in largemouth bass, glucose tolerance test was performed. The findings illustrated in Fig. 2A indicated that the levels of glucose were significantly affected by the time of sampling, dietary treatments, and the interaction between them (*P* < 0.001). HC diet impaired glucose tolerance manifested by a significantly lower area under the curve (AUC), as well as reduced glucose and increased insulin. Consistently, we found that HCA diet significantly ameliorated the insulin sensitivity caused by HC diet, demonstrated by elevated glucose tolerance and insulin, and reduced glucose (Fig. 2A-C). With the increase in the glucose injection time, it can be seen that *ir*, *irs1* and *insulin* presented an overall trend of increasing first and then decreasing, while *pi3kr1* presented obvious decreasing first and then increasing (Fig. 2D). After 1 h of injection, the results indicated that HC diet led to a rise in mRNA level of *ir* and *irs1* and a reduction in mRNA level of *pi3kr1*, and *insulin* expression was not affected by dietary treatments (Fig. 2E). After 3 h following glucose injection, *pi3kr1* mRNA level was increased in HC diet, while the expression of *ir*, *irs1* and *insulin* were not altered (Fig. 2F). At hour 12 after glucose injection, HC diet reduced liver mRNA level of *ir*, *irs1* and *pi3kr1*, while promoted liver mRNA level of *insulin* (Fig. 2G). Surprisingly, HCA diet did not restore these gene expressions in livers at different time during the GTT. Subsequently, we examined the protein levels of insulin resistance markers in the livers. Gray degree analysis showed that HCA diet repressed the accumulation of PTP1B and induced an increase in AKT phosphorylation (Fig. 2H). Real-time PCR analysis indicated that HC diet significantly inhibited the mRNA expression of *pi3kr1* and *insulin* and HCA diet significantly reversed the expression of these genes, whereas *ir* and *irs1* levels remained unchanged (Fig. 2I). The results indicated that astaxanthin has a direct impact on the signaling pathway of PTP1B/PI3K/Akt.

### Astaxanthin altered the hepatic gene expression pattern of largemouth bass

**Fig. 3:**
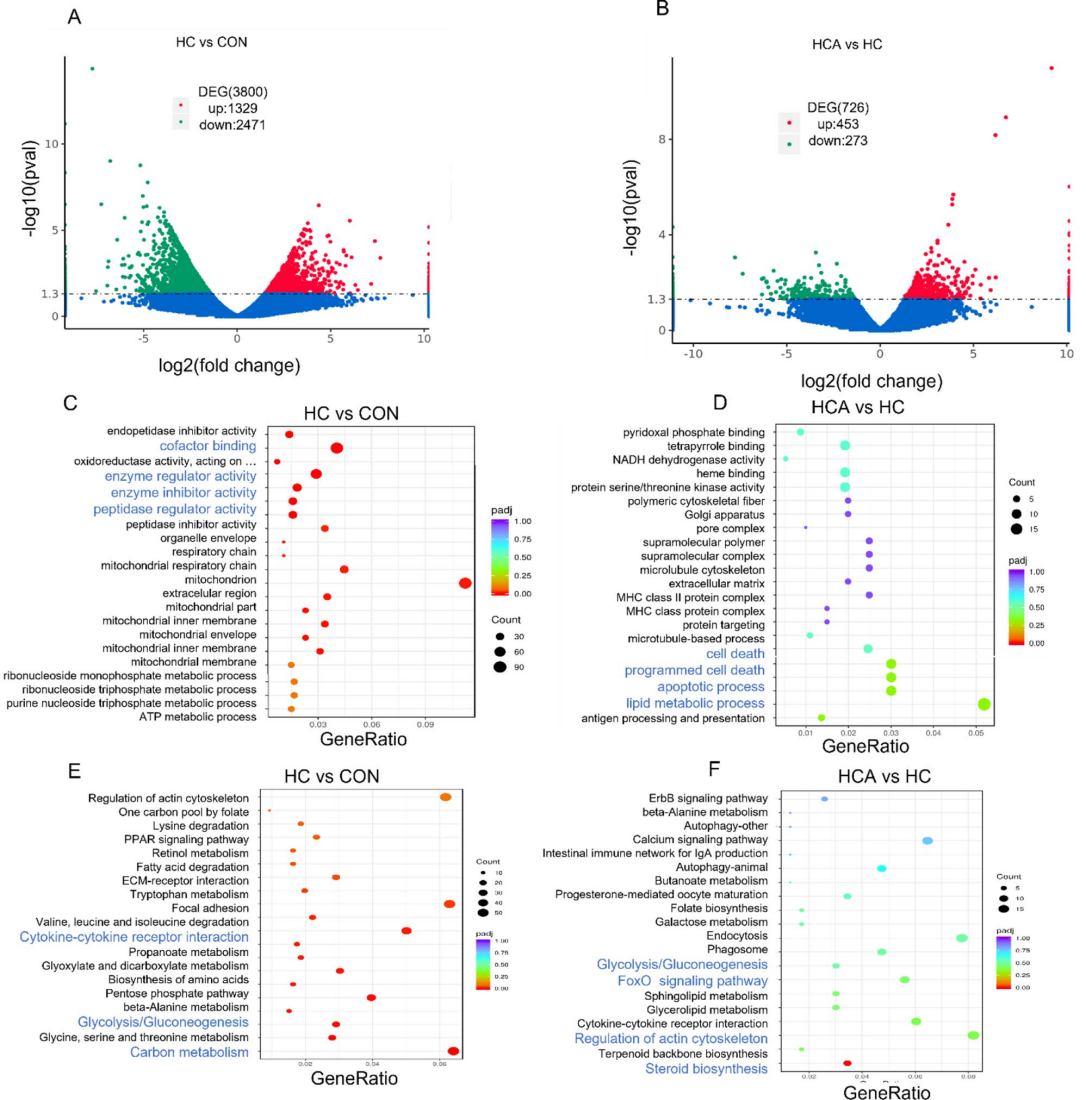
Astaxanthin altered the hepatic gene expression pattern of largemouth bass. (**A**) Volcano plot of differentially expressed genes in HC diet compared with CON diet. Red dots represent upregulated genes and green dots represent downregulated genes. (**B**) Volcano plot of differentially expressed genes in HCA diet compared with HC diet. Red dots represent upregulated genes and green dots represent downregulated genes. (**C**) Bubble plot of Gene Ontology (GO) terms between HC and CON diet. (**D**) Bubble plot of GO terms between HCA and HC diet. (**E**) Bubble plot of KEGG pathways between HC and CON diet. (**F**) Bubble plot of KEGG pathways between HCA and HC diet; CON: control; HC: high-carbohydrate; HCA: high-carbohydrate diet supplemented with astaxanthin

To further elucidate and explain the gene expression patterns of largemouth bass fed with three diets, transcriptome profiles were performed by RNA-seq analysis. By comparison with CON diet, HC diet led to a total 1329 upregulated genes while 2471 downregulated genes (Fig. 3A). By comparison with HC diet, HCA diet led to a total 453 upregulated genes and 273 downregulated genes (Fig. 3B). The Gene Ontology (GO) analysis showed that the differentially expressed genes (DEGs) were highly enriched in cofactor binding, enzyme regulator activity, enzyme inhibitor activity and peptidase regulator activity between HC and CON diets (Fig. 3C). When compared to HC diet, the enriched DEGs were mainly focused on the apoptotic process, cell death programmed cell death and lipid metabolic process in HCA diet (Fig. 3D). KEGG enrichment analysis revealed that DEGs were highly enriched in carbon metabolism, cytokine-cytokine receptor interaction, oxidative phosphorylation and glycolysis/gluconeogenesis between HC and CON diets (Fig. 3E). Furthermore, compared to HC diet, the DEGs were enriched in pathways such as steroid biosynthesis, regulation of actin cytoskeleton, glycolysis/gluconeogenesis and FoxO signaling pathway in HCA diet (Fig. 3F).

### Astaxanthin alleviated liver damage by improving apoptosis, inflammation and oxidative stress in high carbohydrate-fed largemouth bass

**Fig. 4:**
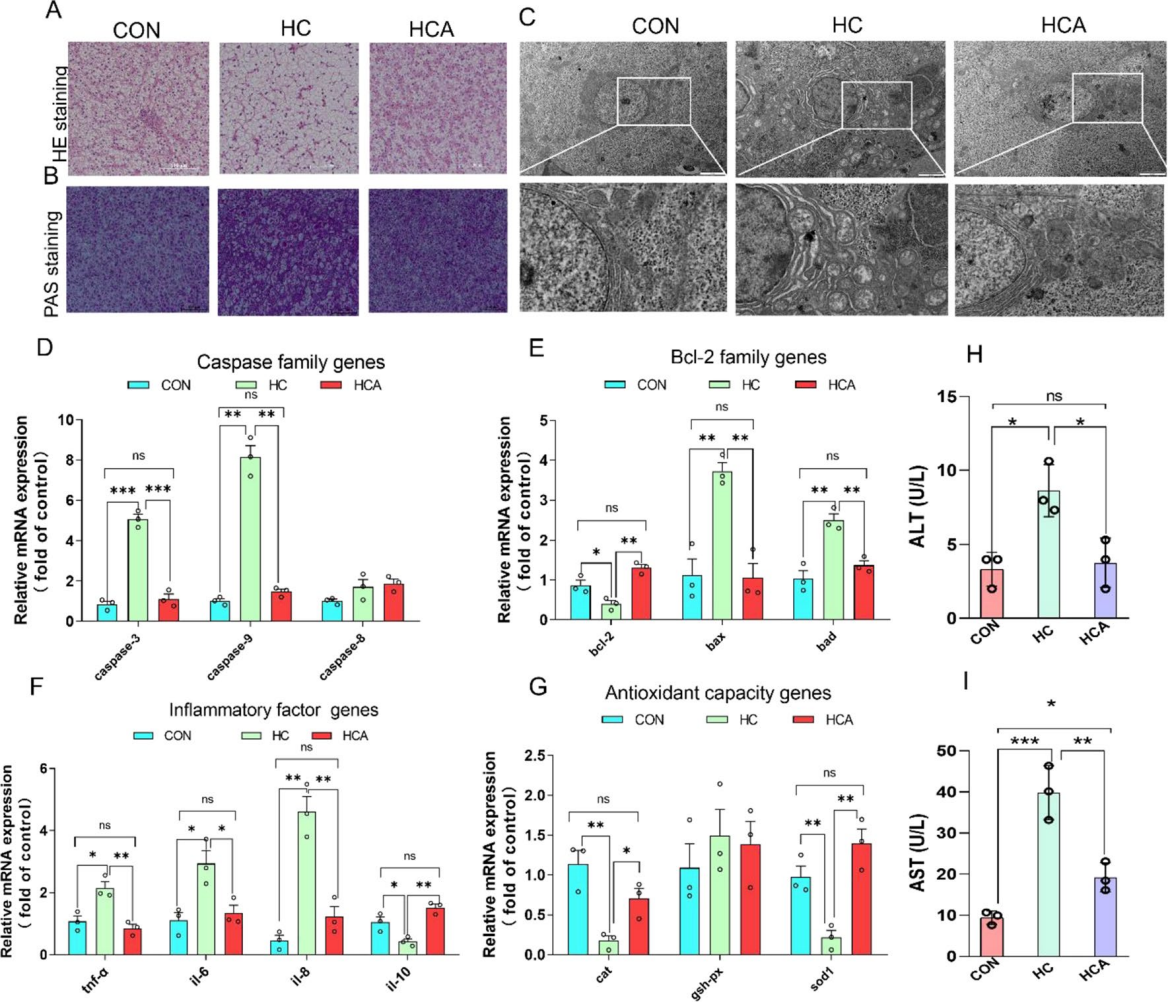
Astaxanthin alleviated liver damage by improving apoptosis, inflammation and oxidative stress in high carbohydrate-fed largemouth bass. (**A**) H&E staining, Scale bar, 100 μm, original magnification×4. (**B**) PAS staining. (**C**) The structure of the ultramicroscopic characteristics and structure in the livers under electron microscopy. (**D**) Relative expression of Caspase family genes (*caspase-3*, *caspase-8* and *caspase-9*) (*n* = 3). (**E**) Relative expression of Bcl-2 family genes (*bcl-2*, *bax* and *bad*) (*n* = 3). (**F**) Relative expression of inflammatory factor genes (*tnf-α*, *il-6*, *il-8* and *il-10*) (*n* = 3). (**G**) Relative expression of antioxidant genes (*cat*, *gsh-px* and *sod1*) (*n* = 3). (**H**) and (**I**), The activities of serum ALT and AST (*n* = 3). Values were mean ± SEM of three biological replicates. AST, aspartate aminotransferase; ALT, alanine aminotransferase. CON: control; HC: high-carbohydrate; HCA: high-carbohydrate diet supplemented with astaxanthin. *P < 0.05, **P < 0.01 and ***P < 0.001; *ns*, no significant difference

The pathology slices stained with H&E and PAS (Fig. 4A and B) showed that the livers of HC-fed largemouth bass exhibited cell swelling, obvious vacuoles, and glycogen accumulation. However, HCA diet alleviated the pathological changes in livers. The obvious mitochondrial damage and the presence of significant amounts of glycogen revealed by transmission electron microscopy (TEM) suggested that HC diet led to a dysfunctional mitochondrion. Notably, the administration of astaxanthin demonstrated a mitigating effect on the mitochondrial damage (Fig. 4C). The qPCR was used to quantify the expression of Caspase family (*caspase-3*, *caspase-8*, and *caspase-9*), Bcl-2 family (*bcl-2*, *bax*, and *bad*), inflammatory factor (*tnf-α*, *il-6*, *il-8*, and *il-10*), and antioxidant capacity (ca.t, g*sh-px*, and *sod1*) genes. As expected, HC diet boosted mitochondrial apoptosis (Fig. 4D and E) and inflammation (Fig. 4F), and decreased antioxidant capacity of livers (Fig. 4G). Correspondingly, astaxanthin has antiapoptotic, anti-inflammatory and antioxidant effects in largemouth bass fed HC diet. In addition, the results also indicated that the increases of serum ALT and AST activities induced by HC diet were reduced by HCA diet (Fig. 4H and I).

### Astaxanthin suppressed HG-induced apoptosis in largemouth bass primary hepatocytes

**Fig. 5:**
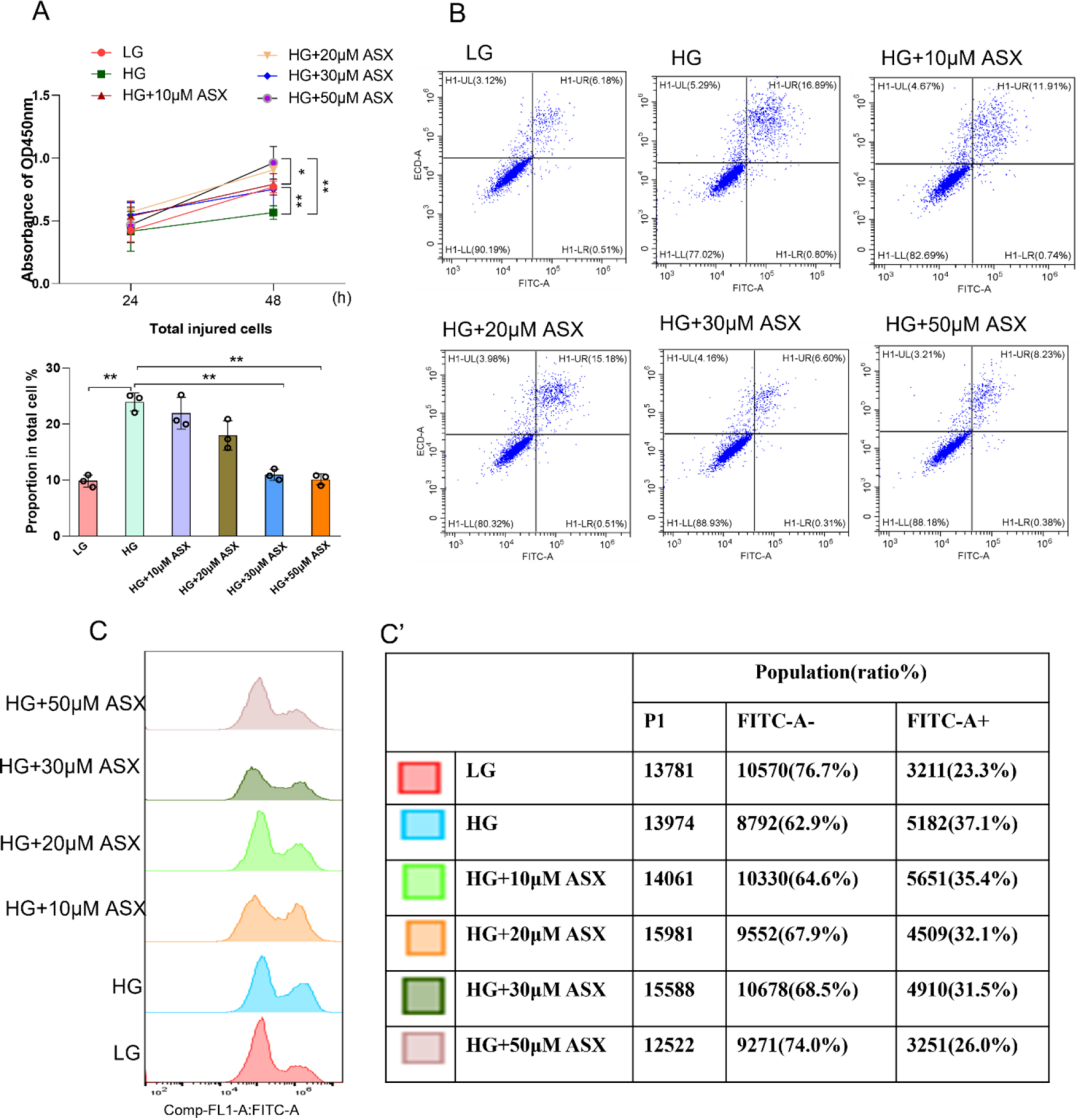
Astaxanthin suppressed HG-induced apoptosis in largemouth bass primary hepatocytes. (**A**) Cell counting kit-8 test (*n* = 3). (**B**) Flow cytometry for apoptosis (*n* = 3), LL: live cells; LR: early apoptotic cells; UR: late apoptotic cells; UL: mechanically damaged cells. (**C**) ROS production analysed by flow cytometry. (**C’**) The proportion of intracellular ROS in primary hepatocytes (*n* = 3). Values were mean ± SEM of three biological replicates. LG: low-glucose; HG: high-glucose. *P < 0.05, **P < 0.01 and ***P < 0.001; *ns*, no significant difference

To further investigate the advantageous mechanism of astaxanthin in largemouth bass, primary hepatocytes were treated with low glucose (LG) or high glucose (HG) conditions, along with varying doses of astaxanthin (10–50 µM). Cell viability was assessed using CCK8 assays (Fig. 5A), revealing that astaxanthin effectively ameliorated the decline in cell viability caused by HG treatment over a 48-h period, with the most significant improvement observed at concentrations of 30 or 50 µM. The proportion of total injured cells was measured using annexin V-FITC/PI staining, it was found that primary hepatocytes treated with 30 or 50 µM astaxanthin exhibited a lower proportion of injured cells compared to those treated with HG **(**Fig. 5B). Flow cytometry was employed to measure ROS production, which demonstrated that HG treatment led to an increase in ROS levels, whereas astaxanthin treatment showed a concentration-dependent decrease in ROS production (Fig. 5C), with the most pronounced effect observed at a concentration of 50 µM. Consequently, further investigation utilized a concentration of 50 µM astaxanthin (named HGA).

### Astaxanthin improved apoptosis induced by high-glucose via p38MAPK/bcl-2/caspase-3 signaling pathway

**Fig. 6:**
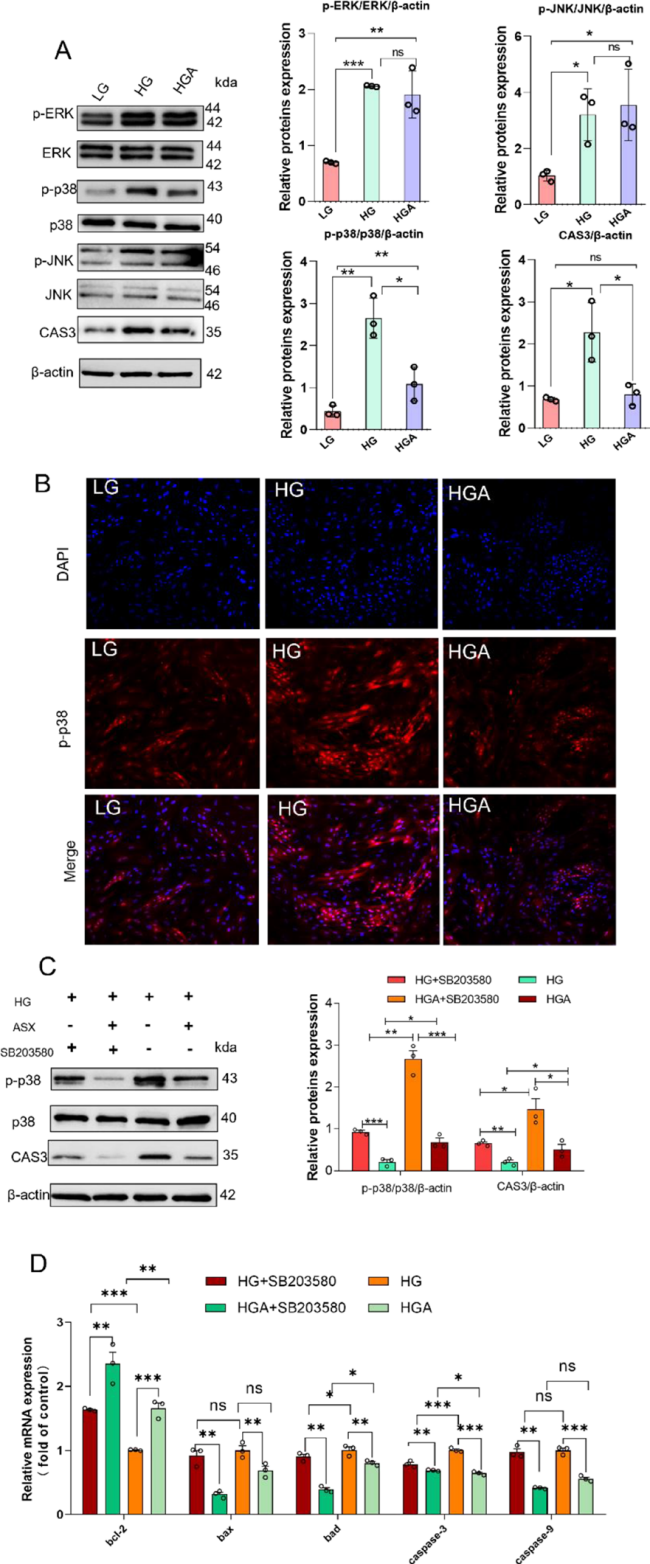
Astaxanthin improved apoptosis induced by high-glucose via p38MAPK/bcl-2/caspase-3 signaling pathway. (**A**) The expression of levels of p-ERK, ERK, p-p38, p38, p-JNK, JNK and CAS3 proteins of primary hepatocytes cultured with three treatments (*n* = 3). (**B**) immunofluorescence for p-p38. (**C**) and (**D**), primary hepatocytes were pretreated with SB203580 for 2 h, inhibitors of the p38MAPK pathways, and treated with HG and HGA for 48 h, respectively. Expression levels of p-p38, p38 and CAS3 were analyzed using western blotting (*n* = 3), expression levels of *bcl-2*, *bax*,* bad*,* caspase-3* and *caspase-9* were analyzed using RT-PCR (*n* = 3). Values were mean ± SEM of three biological replicates. LG: low-glucose; HG: high-glucose; HGA, treated with 50 µM astaxanthin and high-glucose. *P < 0.05, **P < 0.01 and ***P < 0.001; *ns*, no significant difference

In order to elucidate the impact of astaxanthin treatment on the MAPK pathway, western blotting was conducted in vitro model. The findings of this study indicated that HG treatment led to the activation of ERK, JNK, and p38MAPK phosphorylation. Conversely, HGA treatment inhibited the phosphorylation of p38 MAPK, but not ERK and JNK. Additionally, HG treatment resulted in an increase in protein expression of CAS3, whereas HGA treatment blocks this increased protein expression (Fig. 6A). The p-p38 fluorometric assay demonstrated that the heightened fluorescent intensity of p-p38 in HG treatment was reversed by HGA treatment (Fig. 6B). Thus, we ensured that astaxanthin significantly inhibited the p38MAPK signal pathway. In this study, pretreatment with SB203580 (an inhibitor of the p38MAPK signaling pathway) significantly inhibited CAS3 expression at the gene and protein levels (Fig. 6C and D). Furthermore, this effect was significantly enhanced by the addition of astaxanthin. The gene expression levels of *bcl-2* and *bad* were significantly altered in cells treated with HG and HGA, in the presence of SB203580, whereas there were no notable differences observed in the expression of *bax* and *caspase-9*. These findings suggested that astaxanthin may hinder apoptosis induced by high glucose through the p38MAPK/bcl-2/caspase-3 signaling pathway.

## Discussion

Earlier studies have successfully shown the limited use of glucose in largemouth bass, where an excessive intake of carbohydrates had a detrimental impact on their growth and overall health [27, 28]. In this study, the supplementation of astaxanthin led to an improvement of growth performance in largemouth bass fed HC diet. The inclusion of astaxanthin with a concentration of 0.01% had notable beneficial effect on the growth of *Trachinotus ovatus* when fed a high-fat diet [29]. Nevertheless, there was no notable disparity in the developmental progress of *Oncorhynchus mykiss* when exposed to a 0.05% ASX concentration [30]. Variations in dietary patterns, fish species, and concentrations of astaxanthin may contribute to the inconsistent impacts on growth. Further studies for determining the growth-promoting effect of astaxanthin on different fish species may help astaxanthin for aquafeed application.

Our results showed long term intake of a HCA diet improved glucose homeostasis by improving insulin sensitivity, lowering glucose intolerance, and reducing glucose levels. It only taken 4–6 h to return to the baseline blood glucose level after the same dose of glucose injection in omnivorous and herbivorous fish [31], while largemouth bass needed 12 h to return to normoglycemia during a GTT. Further evidence that largemouth bass was a typical sugar intolerance carnivorous fish. Generally speaking, elevated fasting glucose levels are due to hepatic insulin resistance [32]. It appeared that astaxanthin has lycemia-lowering and insulin resistance-improving effects, which was in accordance with the other studies [33]. Specifically, although astaxanthin efficiently regulated the PTP1B/PI3K/Akt signaling cascade in long term HC diet, it failed to alter expression of insulin resistance genes in the liver during the GTT. This discrepancy suggests that different effects of astaxanthin treatment might be related to the length of time for high glucose exposure.

Fish is more likely to convert glucose into glycogen in the liver when fed a high-carbohydrate diet [34]. Red grouper juveniles fed a high carbohydrate diet has reduced growth rate and increased liver glycogen [35]. The increased glycemia observed in many fishes is accompanied by a decline in hepatic glycogen, suggesting stimulation of glycogenolysis and a role in mobilizing carbohydrates [36]. However, in largemouth bass, this is accompanied by elevated glucose and accumulation of glycogen, possibly that is why largemouth bass cannot utilize carbohydrates a metabolic fuel source. In addition, it should be noted that this excessive and irreversible accumulation of liver glycogen can cause glycogenic hepatomegaly, leading to liver dysfunction and liver damage [37]. Astaxanthin has been widely studied and acclaimed as a powerful antioxidant and anti-inflammatory agent under certain pathological conditions [38]. Our results showed that astaxanthin effectively ameliorated liver vacuolization, inflammation, hepatic glycogen deposition and mitochondrial damage induced by the HC diet. This may aid in dealing with the nutritional-technological conundrum associated with producing carnivorous fish feed.

In this study, GO analysis showed that astaxanthin plays an important role in regulating the signaling pathways of apoptosis. Apoptosis consists of two primary routes: the intrinsic pathway, which engages the mitochondria, and the extrinsic pathway, which involves death receptors [39]. Mitochondrial apoptosis is also known as the Bcl-2 signaling pathway [40]. Our findings unequivocally demonstrated that astaxanthin could ameliorate high carbohydrate-induced Bcl-2 signaling pathway by suppressing *caspase-3*, *caspase-9*, *bax*, and *bad* expression and simultaneously restoring *bcl-2* gene expression. The existing scholarly investigations pertaining to the impact of excessive carbohydrate consumption on largemouth bass primarily concentrate on transcript levels, enzyme activity, and metabolites in vivo model [23, 41]. Malwina et al. [42] reported that astaxanthin inhibited cell proliferation by inducing the apoptosis of equine ASC cells by regulating the ratio of Bax/Bcl-2. In this study, we utilized primary hepatocytes cultured in a high glucose setting as a vitro model to evaluate the changes caused by astaxanthin on cell growth and cell death. Our findings also indicated that astaxanthin exhibited a significant ability to enhance primary cell survival and reduce the rate of apoptosis, which was also compatible with what was known of the in vivo model we used.

Under typical cellular circumstances, the production and removal of ROS maintain in a balanced and ever-changing state. However, when the body is stimulated by specific factors, an overproduction of ROS can occur [43]. It is known that mitochondrial damage leads to an increase in ROS production, and that excessive ROS can damage mitochondria significantly more [44, 45]. In this study, we confirmed that that HC diet induced mitochondrial damage in largemouth bass by increased intracellular accumulation of ROS due to decreased expression of antioxidant genes *cat* and *sod1*. Simultaneously, largemouth bass fed HC diet displayed an excessive oxidative stress, which caused a decline in growth performance and liver health. According to our results, astaxanthin modulates oxidative stress within HG treated primary hepatocytes, which evidenced by the observed drop in the number of ROS positive cells and the restoration of the expression of *cat* and *sod1*. Astaxanthin alleviates the adverse effects of high carbohydrate on largemouth bass could also be attributed to its antioxidant property.

The significance of the mitogen-activated protein kinase (MAPK) signaling pathway in apoptosis has been highlighted [46]. This pathway encompasses the ERK, JNK, and p38MAPK pathways, which are known to be crucial in various biological processes such as inflammation, cellular growth, and stress response [47]. In the present investigation, the involvement of MAPK signaling in largemouth bass fed HC diet was examined, and it was found that the phosphorylation of ERK1/2, JNK1/2, and p38MAPK was significantly increased. As a super antioxidant, astaxanthin has been shown to exhibit efficacy in the treatment of diabetic mellitus by suppressing anti-apoptotic activity via modulation of MAPKs and PI3K/Akt pathways [48]. Our observation that astaxanthin significantly inhibited phosphorylation of p38MAPK, but not ERK1/2 and JNK1/2. This result indicated that the mechanism of astaxanthin-inhibited apoptosis might differ from previous studies. Moreover, the present study also demonstrated that astaxanthin may hinder apoptosis induced by high glucose by targeting p38MAPK/bcl-2/caspase-3 signaling pathway. These findings suggest that astaxanthin could be a promising therapeutic target for managing insulin resistance and liver health in carnivorous fish.

## Conclusion

In a word, our findings showed that astaxanthin alleviated high-glucose-induced mitochondrial apoptosis in largemouth bass via the regulation of p38 MAPK/bcl-2/caspase-3 pathway. To our knowledge, astaxanthin reduces cell apoptosis, ameliorates oxidative stress and mitochondrial damage. This study provides strong evidence for the role of astaxanthin in fish metabolic syndrome prevention and treatment. Besides, astaxanthin is first shown to improve insulin resistance through the PTP1B/PI3K/Akt axis, which promotes the use of astaxanthin in aquafeeds and provide a potential strategy to improve the utilization of dietary carbohydrate in carnivorous fish.

## Electronic supplementary material

Below is the link to the electronic supplementary material.

Supplementary Material 1

Supplementary Material 2

## Data Availability

No datasets were generated or analysed during the current study.

## Abbreviations

acadm: acyl-CoA dehydrogenase medium
AKT: Protein kinase B
ALT: Alanine transaminase
AMPK: Adenosine 5‘-monophosphate (AMP)-activated protein kinase
AST: Aspartate transaminase
ASX: Astaxanthin
bcl-2: B-cell lymphoma-2
bad: BCL2 associated agonist of cell death
bax: BCL-2-associated X protein
BW: Body weight
cat: Catalase
CCK8: Cell counting kit-8
CF: Condition factor
CON: Control
cytb: Cytochrome b
DMEM: Dulbecco modified Eagle medium
EDTA: Ethylenediaminetetraacetic acid
ef-1α: Elongation factor 1α
enpp1: Phosphodiesterase 1
FBS: Fetal bovine serum
FITC: Fluorescein isothiocyanate
gsh-px: Glutathione peroxidase
GSK3β: Glycogen synthase kinase 3β
GTT: Glucose tolerance test
H&E: Hematoxylin and eosin
HSI: Hepatosomatic index
ir: Insulin receptor
ir-β: Insulin receptorβ
irs1: Insulin receptor substrate1
il-6: Interleukin-6
il-8: Interleukin-8
il-10: Interleukin-10
MAPK: Microtubule-associated protein kinase
p-Akt: Phosphorylation of protein kinase B
PAS: Periodic acid-schiff
PBS: Phosphate-buffered saline
PI: Propidium iodide
PI3K: Phosphoinositide 3-kinase
PTP1B: Protein tyrosine phosphatase-1B
PVDF: Polyvinylidene fluoride
qRT-PCR: Quantitative real-time PCR
ROS: Reactive oxygen species
SGR: Specific growth rate
sod1: Superoxide dismutase 1
TEM: Transmission electron microscopy
VSI: Viscerosomatic index
WG: Weight gain
