# Supplementary material for: Astaxanthin attenuates glucose-induced liver injury in largemouth bass: role of p38MAPK and PI3K/Akt signaling pathways
Source: Cell Biosci. 2024 Sep 19;14:122. doi: 10.1186/s13578-024-01304-7 (PMC11414117; doi:10.1186/s13578-024-01304-7)
Supplement: Supplementary file 1 — Supplementary Material 1 [file 13578_2024_1304_MOESM1_ESM.docx]

**Experimental diet:** All ingredients were ground into powder and mixed thoroughly by a feed mixer (A-200T Mixer Bench Model unit, Resell Food Equipment Ltd, Ottawa, Canada). A screw-press pelletizer was used to obtain 2.0 mm pellets from the mixture containing fish oil, soy oil, and soya lecithin and water (F-26, South China University of Technology, Guangzhou, China). Pellets were dried at 16 °C in a well-ventilated condition until moisture content dropped below 10%, and then stored at -20 °C.

**Experiment design and feeding management:** To acclimate to the experimental conditions, all fish were raised in a circulating culture system and feed with a commercial diet (Tongwei Co., Ltd., China) for two weeks. 360 juvenile largemouth bass of uniform weight (average initial weight, 8.24±0.01 g) were chosen and irregularly distributed to 12 tanks (100 L) in quadruplicate (30 fish per tank). In the feeding trial, largemouth bass were fed twice a day at 9:00 and 17:00 for eight weeks to satiation. During the 8-week trial, fish were reared in a recirculating water system. The water temperature was kept between 25 and 28 ℃, while the dissolved oxygen at about 9.0 mg L^-1^, pH varied from 7.9 to 8.2, and ammonia nitrogen level lower than 0.2 mg L^-1^.

**Growth performance calculation:** Here were the parameters used to calculate growth performance and morphology:

Weight gain (WG, %) = 100× (final fish weight−initial fish weight)/Initial fish weight;

Survival rate (%) = 100× final number of fish/initial number of fish;

Specific growth rate (SGR, % day^-1^) = 100× [ln (final body weight) −ln (initial body weight)] /feeding days;

Condition factor (CF, g cm^-3^) = 100×body weight/body length^3^;

Viscerosomatic index (VSI, %) = 100× (viscera weight/body weight);

Hepatosomatic index (HSI, %) = 100× (liver weight/body weight).

**Glucose tolerance test (GTT):** 30 fish from each diet were lightly anesthetized with MS-222 (200 mg/L). To minimize the deviation of plasma glucose levels, fish were given an intraperitoneal injection of glucose [1 g glucose/kg BW] as quick as possible. The fish were randomly placed in three tanks at a rate of six fish per tank, and samples were taken at 1, 3, 6 and 12 h. To minimize stress associated with sampling, one tank of fish was sampled for each sampling time. Blood was immediately collected from the caudal vein, centrifuged for 10 min at 4°C to obtain serum, and then stored at -80 °C until analysis.

**Transcriptomic analysis:** a paired-end RNA-seq sequencing library was prepared and sequenced using the Illumina platform, following quantification with the Qubit 2.0 Fluorometer. Differential expression analysis of two treatments (three biological replicates per treatment) was performed using the DESeq2 R package (1.20.0). Gene Ontology (GO) enrichment analysis of differentially expressed genes was implemented by the cluster Profiler R package, in which gene length bias was corrected. GO terms with corrected P value less than 0.05 were considered significantly enriched by differential expressed genes. KEGG is a database resource for understanding high-level functions and utilities of the biological system, such as the cell, the organism and the ecosystem, from molecular-level information, especially large-scale molecular datasets generated by genome sequencing and other high-through put experimental technologies (http://www.genome.jp/kegg/). We used cluster Profiler R package to test the statistical enrichment of differential expression genes in KEGG pathways.

**Antibodies used for western blotting assay:** The primary antibodies used included anti-β-actin (4970S; Cell Signaling Technology, USA, diluted 1:1000), anti-Akt (8200S; Cell Signaling Technology, USA, diluted 1:1000), anti-phospho-Akt (8200S; Cell Signaling Technology, USA, diluted 1:1000), anti-PTP1B (11334; Proteintech, USA, diluted 1:1000), anti JNK1 (3708S; Cell Signaling Technology, USA, diluted 1:1000), anti p-SAPA/JNK(4668T; Cell Signaling Technology, USA, diluted 1:1000), p38 MAPK (9212S; Cell Signaling Technology, USA, diluted 1:1000), Phospho-p38 MAPK (4511T; Cell Signaling Technology, USA, diluted 1:1000), p44/42 MAPK (4695T; Cell Signaling Technology, USA, diluted 1:1000), Phospho- p44/42 MAPK (4370T; Cell Signaling Technology, USA, diluted 1:1000) and CAS3 (T40044F; Abmart, China, diluted 1:500).

**Immunofluorescence analysis:** Treated cells were washed with cold TBST, and fixed in 4% paraformaldehyde (cat. no. AAPR12), permeabilized with 0.1% Triton X-100 (cat. no. AAPR96) and blocked with 2% BSA (cat. no. AAPR305) at 4 ˚C overnight. The cells were washed three times and incubated with Phospho-p38 MAPK antibody (dilution 1:100) and the corresponding secondary antibody (Alexa Fluor goat anti-rabbit 594, 1:100). ProLong Gold Antifade (cat. no. P36941) was plated in the laser confocal culture dish to stain the nucleus and prevent the fluorescence quenching. The slips were imaged using a Leica SP8 STED confocal laser scanning microscope (Leica Microsystems GmbH).
