# Supplementary figures and images for: Astaxanthin attenuates glucose-induced liver injury in largemouth bass: role of p38MAPK and PI3K/Akt signaling pathways
Source: Cell Biosci. 2024 Sep 19;14:122. doi: 10.1186/s13578-024-01304-7 (PMC11414117; doi:10.1186/s13578-024-01304-7)

Fig.2H

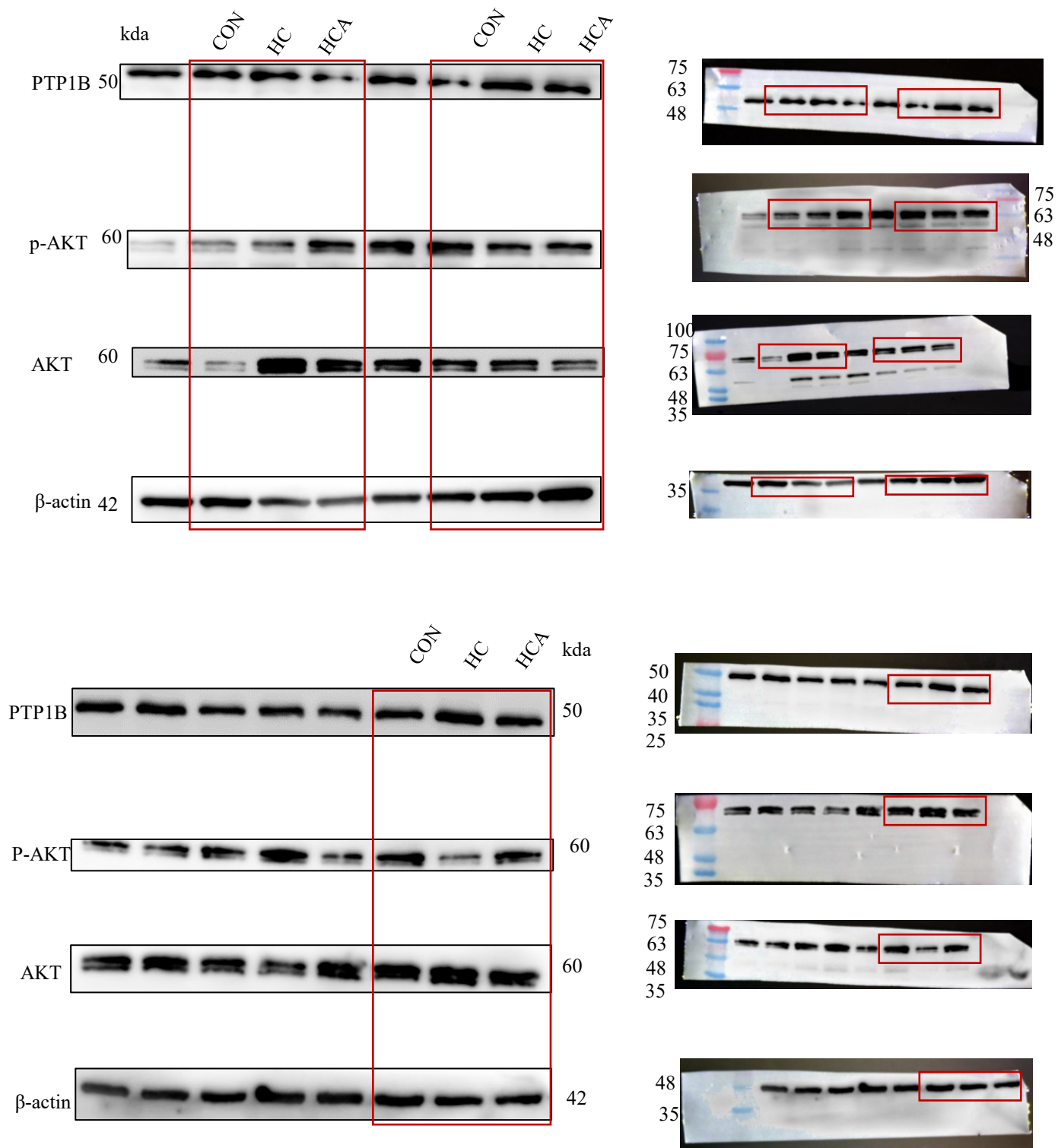

Fig.6A

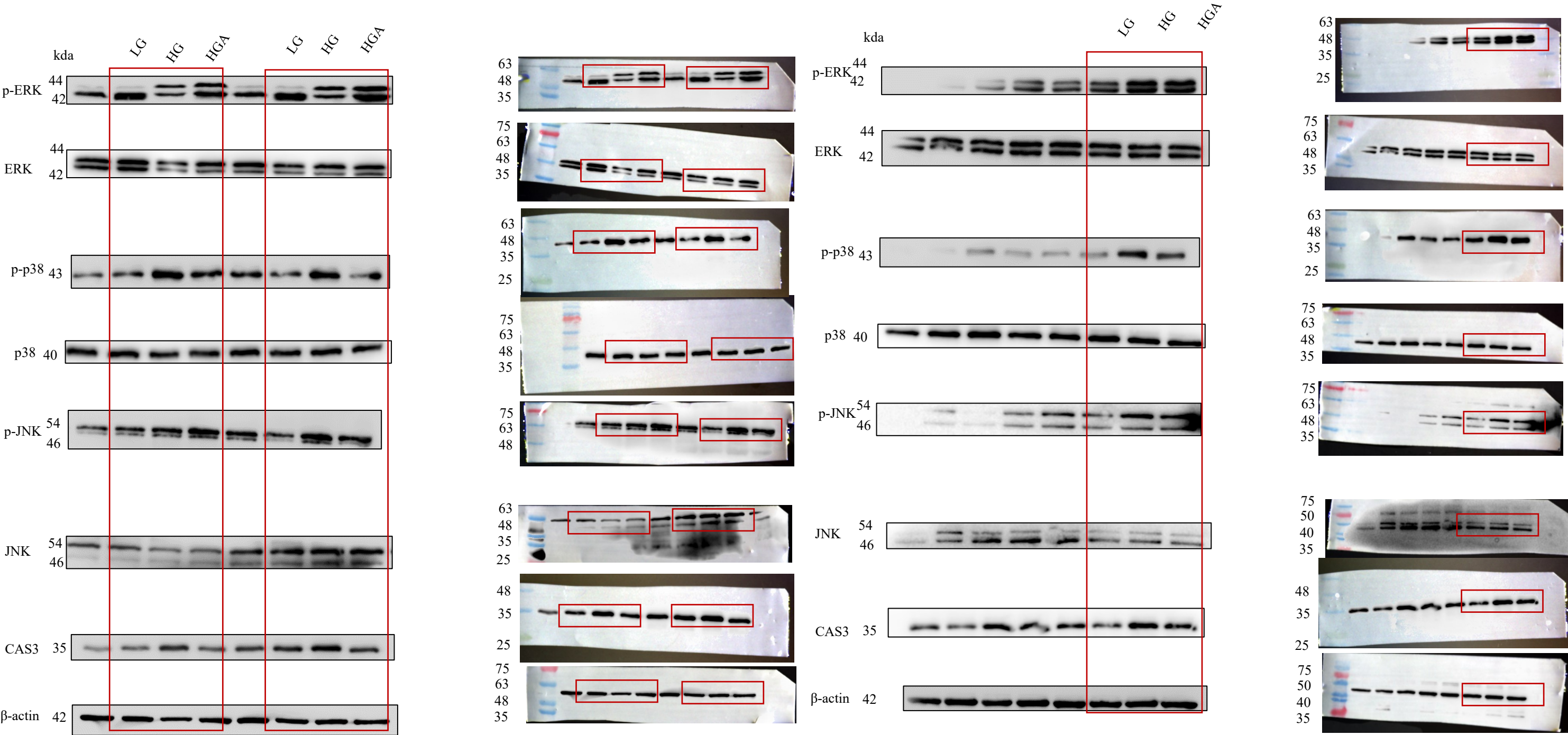

Fig.6C

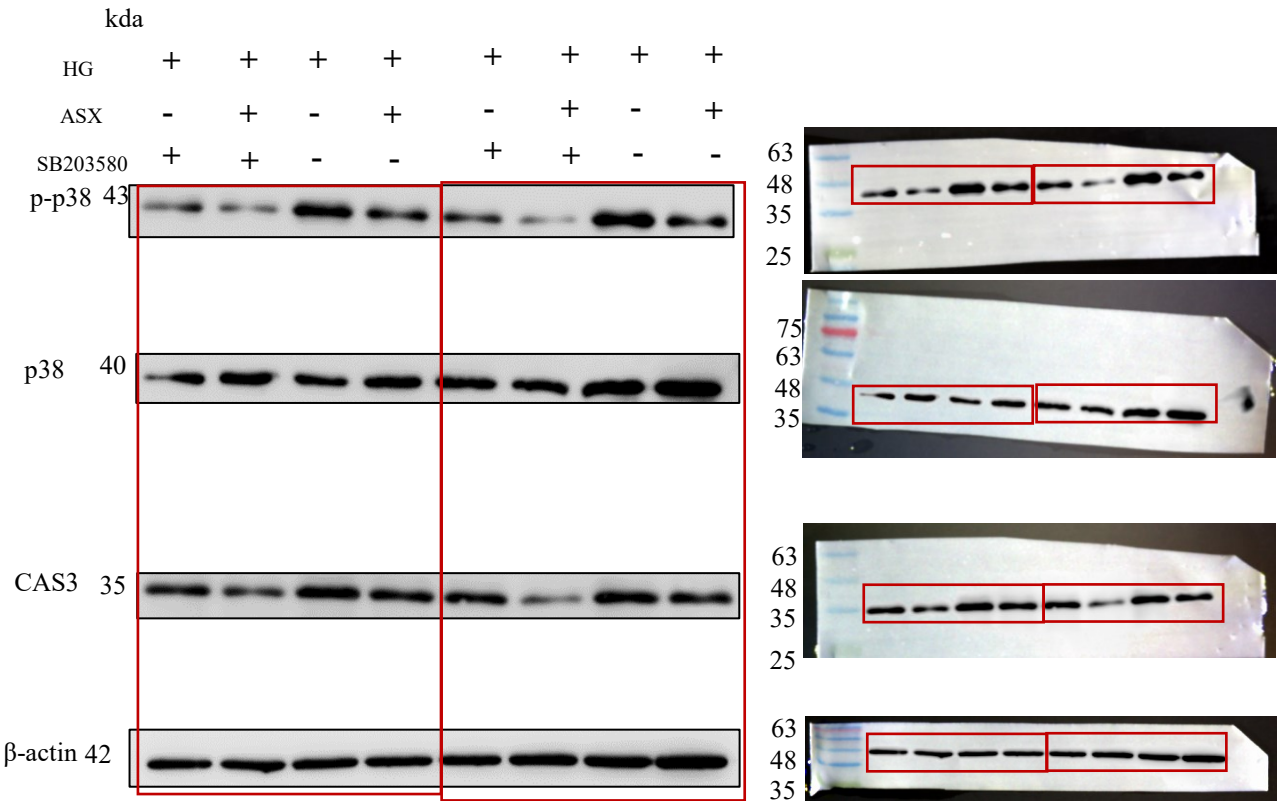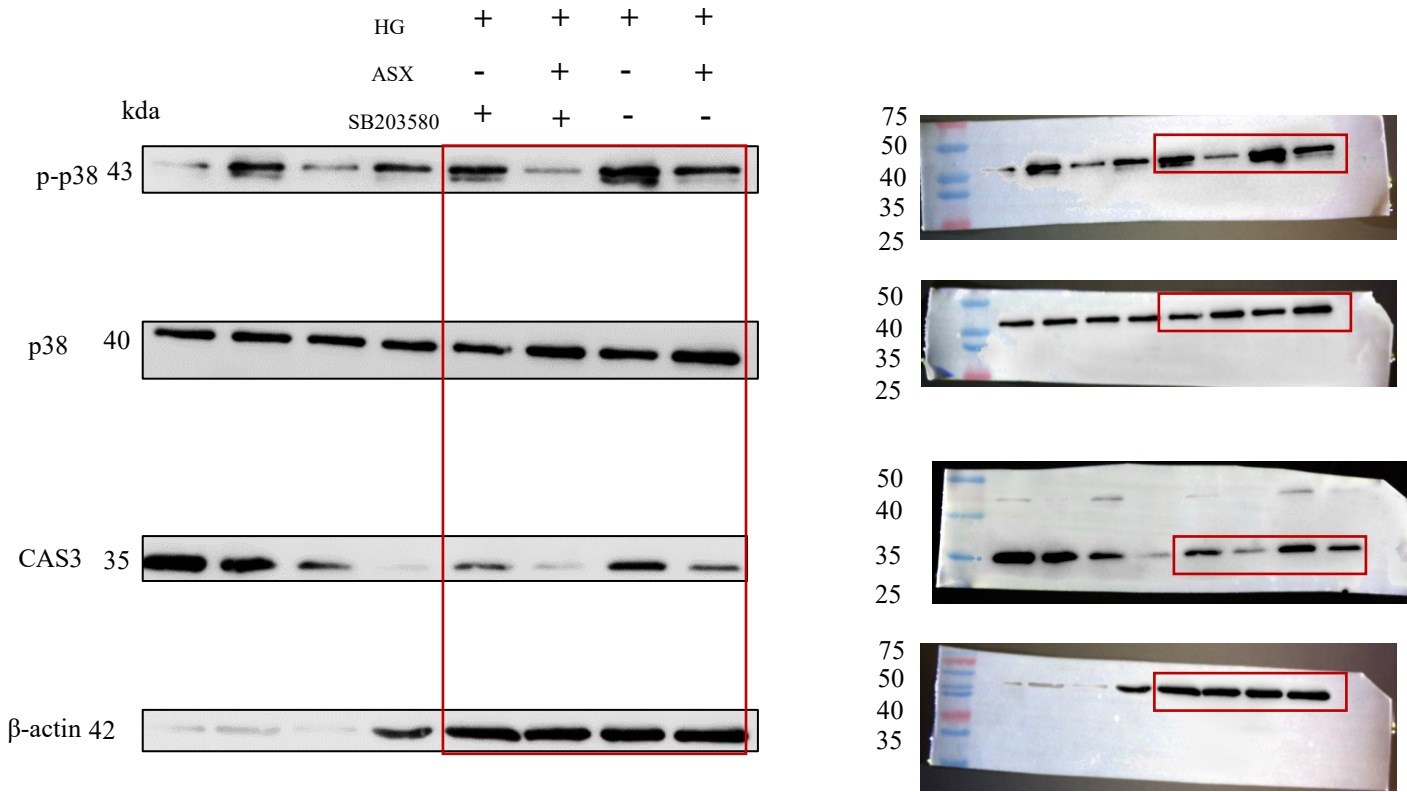

Supplement: Supplementary file 2 — Supplementary Material 2 [file 13578_2024_1304_MOESM2_ESM.pdf]
